# Optimizing Tensile Strength of Low-Carbon Steel Shafts with Stacked Ring Substrates in WAAM Using Taguchi and Random Forest Regression

**DOI:** 10.3390/ma18225065

**Published:** 2025-11-07

**Authors:** Van-Minh Nguyen, Pham Son Minh, Minh Huan Vo

**Affiliations:** 1 Faculty of Mechanical Engineering, Ho Chi Minh City University of Technology and Education, Ho Chi Minh City 71307, Vietnam; minhps@hcmute.edu.vn; 2 Faculty of Electrical and Electronic Engineering, Ho Chi Minh City University of Technology and Education, Ho Chi Minh City 71307, Vietnam; huanvm@hcmute.edu.vn

**Keywords:** Wire Arc Additive Manufacturing (WAAM), tensile strength, Taguchi L25 orthogonal array, process parameter optimization, step length, random forest regression, MIG Welding

## Abstract

Wire Arc Additive Manufacturing (WAAM) enables cost-effective fabrication of complex metallic components but faces challenges in achieving consistent tensile strength for cylindrical parts with intricate internal features (e.g., cooling channels, helical grooves), where conventional machining is often infeasible or prohibitively expensive. This study introduces a novel stacked ring substrate strategy with pre-formed low-carbon steel rings defining complex internal geometries, followed by external WAAM deposition using ER70S-6 wire to overcome these limitations. Five process parameters (welding current: 110–130 A; offset distance: 2.5–3.0 mm; Step Length: rotary to straight; torch speed: 400–500 mm/min; weld thickness: 2.0–3.0 mm) were optimized using a Taguchi L25 orthogonal array (25 runs in triplicate). ANOVA identified Step Length as the dominant factor, with straight paths significantly reducing thermal cycling and improving interlayer bonding, alongside a notable current × speed interaction. Optimal settings achieved tensile strengths of 280–290 MPa, significantly below wrought ER70S-6 benchmarks (400–550 MPa) due to interfacial weaknesses at ring fusion zones and thermal accumulation from stacked cylindrical geometry, a limitation acknowledged in the absence of microstructural or thermal history data. A Random Forest Regressor predicted strength with R^2^ = 0.9312, outperforming conventional models. This hybrid approach significantly enhances design freedom and mechanical reliability for high-value cylindrical components in aerospace and tooling, establishing a scalable, data-driven framework for geometry-constrained WAAM optimization.

## 1. Introduction

Wire Arc Additive Manufacturing (WAAM) is an advanced additive manufacturing technique that has garnered significant interest for its ability to produce large-scale, complex metallic components with high deposition rates and cost efficiency compared to other methods, such as laser powder bed fusion or electron beam melting [1,2,3]. WAAM employs an electric arc as the heat source and a metal wire as the feedstock, typically utilizing processes like Gas Metal Arc Welding (GMAW), Cold Metal Transfer (CMT), or Tungsten Inert Gas (TIG) welding [4,5]. This approach is particularly well suited for fabricating components from materials such as stainless steel, aluminum, titanium, and nickel-based alloys, making it applicable to industries including aerospace, automotive, and marine [6,7,8,9]. The strength of WAAM lies in its capacity to create near-net-shape parts with minimal material waste and reduced lead times, positioning it as a viable alternative to conventional subtractive manufacturing [8,10,11].

Among compatible alloys, ER70S-6 low-carbon steel is a preferred choice in WAAM due to its excellent arc stability, weldability, and mechanical reliability. With a controlled composition of 0.06–0.15% C, 1.40–1.85% Mn, and 0.80–1.15% Si, this wire minimizes defect formation and supports consistent microstructural development during multi-layer deposition [12,13,14,15]. With a nominal composition of 0.06–0.15% carbon, ER70S-6 mild steel wire is favored for its ability to produce components with consistent microstructures and minimal defects when process parameters are optimized [14,16]. However, mechanical performance is highly dependent on process parameters including welding current, torch speed, wire feed rate, interlayer offset, deposition path, and layer thickness which regulate heat input, cooling dynamics, bead geometry, and residual stress accumulation [2,11,17,18]. These variables collectively influence grain refinement, phase distribution, and interlayer bonding, necessitating rigorous optimization to ensure structural integrity [3,19,20].

Substantial research has focused on parameter optimization in WAAM. Vora et al. (2022) demonstrated that controlled welding current and torch speed in GMAW-based WAAM of SS316L yield tensile strengths of 500–600 MPa through improved bead uniformity and reduced porosity [21,22]. Chen et al. (2017) reported enhanced strength in WAAM 316L relative to cast equivalents, attributed to dendritic refinement under moderate thermal input [23,24]. For low-carbon steels, Dekis et al. (2025) achieved 400–550 MPa in flat ER70S-6 builds, identifying torch speed and heat input as dominant factors in grain size control [12]. Frazier (2014) and DebRoy et al. (2018) highlighted the role of deposition strategy and wire feed synchronization in mitigating thermal cycling and defect propagation, particularly in multi-pass structures [25,26]. DebRoy et al. (2018) further emphasized that parameters such as wire feed rate and deposition strategy influence microstructural evolution and defect formation, impacting the mechanical integrity of WAAM parts [26,27]. These investigations underscore the necessity of systematic parameter control to achieve reproducible mechanical properties in WAAM [28,29].

Despite progress in planar geometries, cylindrical components with complex internal architectures present distinct challenges. Curved deposition paths introduce non uniform heat dissipation, elevated thermal gradients, and increased susceptibility to distortion, cracking, or delamination [5,23,30]. Applications in pressure vessels, hydraulic shafts, and mold tooling frequently require intricate internal features such as cooling channels, helical grooves, or hollow cores that are technically infeasible or economically prohibitive to produce via conventional machining due to limited tool access and high material removal [31,32]. Although advanced path planning and interlayer cooling have improved cylindrical WAAM [5,31,33], internal geometric complexity remains a critical barrier, restricting design innovation and industrial scalability.

This study introduces a novel stacked ring substrate strategy to address this limitation. Pre-fabricated low-carbon steel (CT3) rings are assembled to define complex internal profiles prior to WAAM deposition, enabling features unattainable through external layering alone. External cylindrical walls are subsequently built using ER70S-6 wire, forming a hybrid structure that combines the precision of pre-forming with the versatility of additive manufacturing. This approach reduces machining demands, enhances geometric freedom, and distributes thermal load across ring interfaces, potentially mitigating distortion in curved builds [32,34].

To optimize tensile strength within this configuration, five process parameters were selected based on their established influence on heat input, bead overlap, and thermal history welding current, offset distance, step length, torch speed, weld thickness. Parameter ranges were constrained to maintain heat input within 188–550 J/mm, a window known to promote fine grained ferrite while minimizing coarse Widmanstätten structures and excessive residual stress [12,14]. A Taguchi L25 orthogonal array was implemented to evaluate main effects and two-way interactions with high statistical efficiency, requiring only 25 experimental runs (in triplicate) versus 3125 for a full factorial design [35,36,37]. Analysis of Variance (ANOVA) with partial eta squared was applied to rank parameter contributions and detect significant interactions.

Complementing classical design of experiments, a Random Forest Regressor (RFR) was developed to model non-linear parameter–property relationships and support predictive optimization. While machine learning has been integrated with ANOVA in additive manufacturing [38], its application to cylindrical WAAM of low-carbon steel using stacked ring substrates remains largely unexplored [12,14,31,39]. The specific objectives of this investigation are:

- To quantify the individual and interactive effects of five WAAM process parameters on tensile strength using Taguchi L25 design and ANOVA.
- To identify optimal parameter settings that maximize mechanical performance in cylindrical stacked ring substrates.
- To develop and validate a Random Forest Regression model for high-accuracy prediction of tensile strength, enabling data-driven process control.
- To elucidate the influence of deposition path strategy (Step Length) on thermal management and mechanical integrity, establishing a foundation for future microstructural and thermographic validation.

This integrated experimental and computational framework aims to advance WAAM process optimization for complex cylindrical geometries, providing a scalable methodology for industrial applications requiring both structural performance and geometric intricacy.

## 2. Materials and Methods

### 2.1. Materials

The feedstock material for this study was ER70S-6 low-carbon steel wire, chosen for its excellent weldability, cost-effectiveness, and robust mechanical properties, making it ideal for Wire Arc Additive Manufacturing (WAAM) applications. The wire, with a diameter of 1.2 mm, had a chemical composition of 0.06–0.15% carbon, 1.40–1.85% manganese, 0.80–1.15% silicon, ≤0.035% phosphorus, ≤0.025% sulfur, and ≤0.50% copper [14]. This composition ensures minimal defect formation, such as porosity or inclusions, and promotes a consistent microstructure during deposition, suitable for structural components like cylindrical shafts [21,40]. The substrates were pre-fabricated as stacked low-carbon steel (CT3) rings, enabling complex internal geometries (e.g., hollow sections, spiral channels) that are difficult to achieve via conventional machining. This innovative stacked ring configuration enables the creation of complex internal profiles, such as hollow or variable cross-sectional structures, which are challenging or economically unfeasible to produce using conventional methods like forging or casting [41]. This approach enhances WAAM’s applicability for high-performance industries, including aerospace, automotive, and marine, by addressing challenges related to anisotropy and residual stresses [6,19].

### 2.2. Specimen Fabrication

#### 2.2.1. Specimen Design

**Figure 1:**
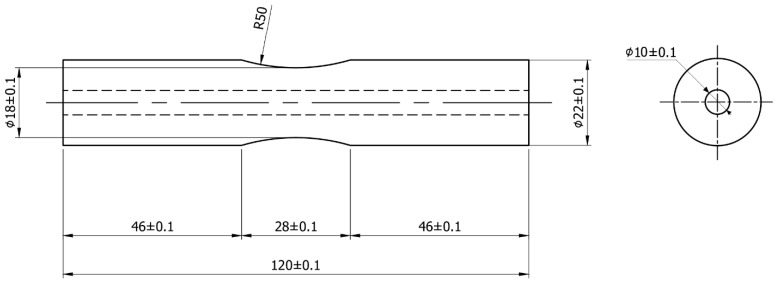
Technical Drawing of Hourglass-Shaped Specimen for Tensile Testing.

The specimens were designed as cylindrical components with an hourglass configuration, selected to concentrate bending stresses at the gauge section during fatigue testing, in accordance with TCVN standards [42], as shown in Figure 1.

#### 2.2.2. WAAM Fabrication Setup

**Figure 2:**
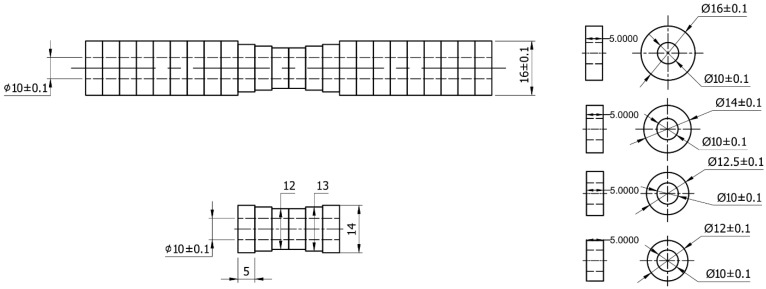
Design of Stacked Ring Substrate Arrangement for WAAM Fabrication.

Cylindrical shaft specimens were fabricated by depositing successive steel rings onto a base plate, as illustrated in Figure 2, with each layer offset to form a stacked structure. This technique enables the creation of complex internal profiles, such as hollow features or cooling channels, by securing the rings through Metal Inert Gas welding (MIG) in a layer-by-layer process. The rings, made of low-carbon steel (CT3) compatible with the GEMINI-GM70S welding wire (GEMINI, Samut Prakan City, Thailand), were stacked to form the standard specimen. The substrate consisted of nine rings (diameter 16 mm) at both ends. The central section included rings with diameters of 14–12 mm, arranged to meet the specimen’s design specifications (Figure 2).

**Figure 3:**
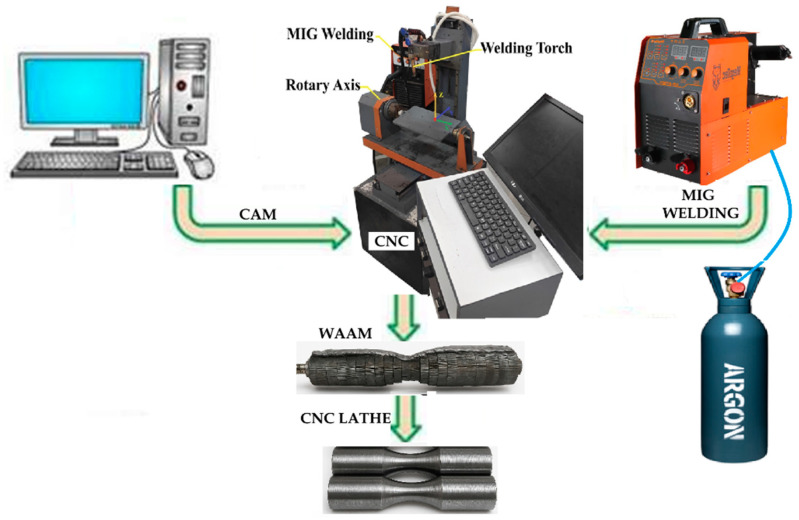
Schematic of 4-Axis CNC WAAM System for ER70S-6 Stacked Ring Substrates.

The deposition process utilized either a rotary Step Length, where the substrate rotated under a stationary torch, or a straight Step Length, involving linear torch movement, to investigate the impact of Step Length on mechanical properties [39]. Inter-layer cooling pauses were implemented to manage thermal gradients and reduce residual stresses, mitigating challenges such as distortion or cracking in cylindrical builds [6,19]. The fabrication was performed using a TEA 4-axis CNC machine WAAM system (TAE-Vietnam, Ho Chi Minh City, Vietnam) integrated with a Gas Metal Arc Welding (GMAW) machine unit model Jasic MIG-270 (Jasic Technology, Shenzhen, China), equipped with a MIG welding torch [20,43]. A wire feeder ensured controlled feedstock delivery, and an Argon shielding gas system, maintained at a flow rate of 8–10 L/min, stabilized the arc, protected the weld pool from atmospheric contamination, and minimized oxidation [1]. The welding voltage was fixed at 20 V to regulate heat input and ensure stable droplet transfer, reducing issues such as spatter or porosity that could compromise component quality [22]. Figure 3 illustrates the schematic of the WAAM setup.

**Figure 4:**
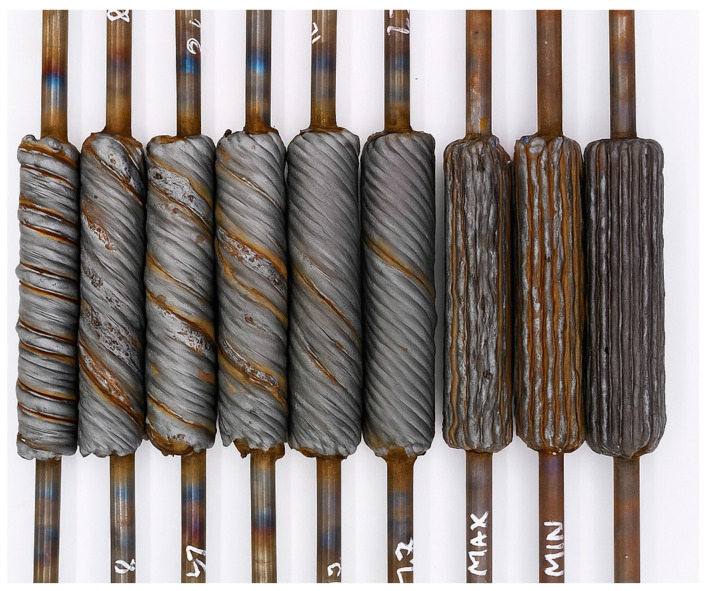
Rough-Built Specimen After WAAM Fabrication.

Post-deposition, the specimens were inspected for material deficiencies and cracks, as shown in Figure 4.

**Figure 5:**
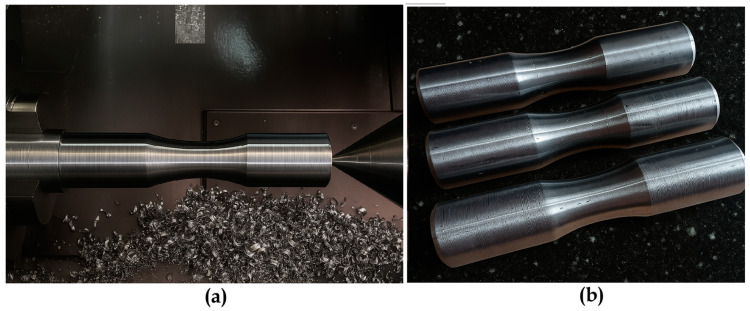
(**a**) Specimen During CNC Lathe Post-Processing, (**b**) Specimen After Post-Processing.

After WAAM, the rough-built components were machined on a CNC lathe to achieve precise dimensions conforming to TCVN standards [42] for tensile testing, with a gauge length of 28 mm and a diameter of 18 mm to ensure uniformity and consistency [2]. This machining process removed surface irregularities and weld imperfections, ensuring a smooth surface to minimize stress concentrations during tensile testing. Lathe machining was carefully controlled to maintain the concentricity of the stacked steel rings, preserving the structural integrity of the shaft while achieving the external dimensions specified in the fatigue sample design (Figure 5).

### 2.3. Experimental Design

A Taguchi L25 orthogonal array was employed to systematically evaluate the effects of five critical process parameters on the tensile strength of WAAM-fabricated shaft components: welding current (I, A), offset distance (mm), Step Length (a, mm), torch speed (mm/min), and weld thickness (α, mm). Each parameter was varied across five levels, as detailed in Table 1, to explore a comprehensive range of conditions while minimizing experimental runs [35]. The Taguchi method was selected for its efficiency in identifying significant factors and their interactions with a reduced number of experiments, ensuring statistical robustness [37].

The selection of these five process parameters—welding current, offset distance, Step Length, torch speed, and weld thickness was driven by their significant influence on heat input, weld bead geometry, and microstructural evolution in WAAM using ER70S-6 low-carbon steel, as established in prior studies [1,12,13]. Parameter ranges were selected to maintain heat input (HI) within an optimal range of 188–550 J/mm for ER70S-6 WAAM, calculated using the formula: HI (J/mm) = (I × Volt × 60)/V, with a fixed arc voltage of 20 V (typical for MIG ER70S-6 welding) [12,13,14]. This HI range promotes refined ferrite microstructures, minimizes coarse grain formation, and enhances tensile strength by controlling cooling rates and reducing thermal accumulation.

**Figure 6:**
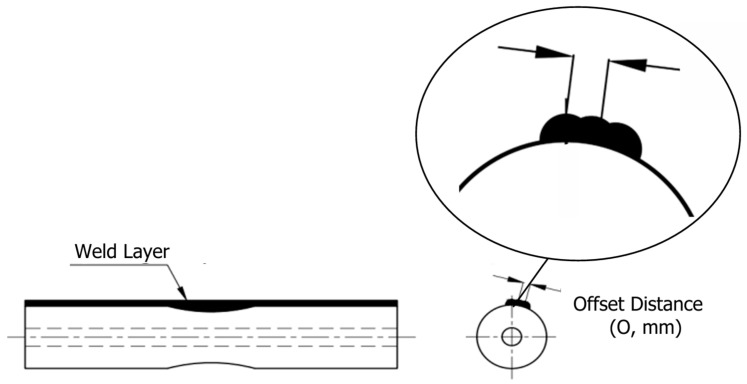
Illustration of Offset Distance in WAAM Weld Bead Deposition.

**Figure 7:**
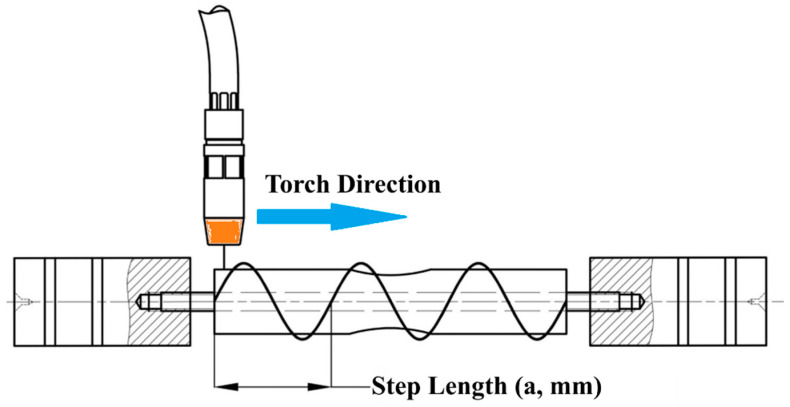
Rotary and Straight Deposition Path Strategies in WAAM.

Welding current (110–130 A) modulated heat input, directly affecting fusion and bead density [27,44,45], while torch speed (400–500 mm/min) controlled cooling rates and grain structure [12,46]. Offset distance (2.5–3.0 mm, see Figure 6) and weld thickness (2.0–3.0 mm) optimized bead overlap [22,47], minimizing defects such as porosity or cracking [13]. Step Length, ranging from rotary to straight ((illustrated in Figure 7), addressed the unique thermal and interlayer bonding challenges of the novel stacked ring geometry, which requires precise control to reduce residual stresses and ensure structural integrity [12,19,20]. To mitigate aliasing inherent in the L25 design particularly for higher-order effects—Step Length was numerically encoded (0 mm = rotary, 1000 mm = straight), approximating path linearity and enabling quantitative comparison. Sensitivity analysis confirmed robustness of significance and contribution estimates under this encoding scheme [32]. Five levels per parameter were chosen to fit the Taguchi L25 orthogonal array, enabling efficient exploration of the parameter space, capturing non-linear effects, and evaluating interactions with minimal experimental runs (25 runs compared to 3125 for a full factorial design) [35].

Experiments were conducted in triplicate for each parameter combination to enhance statistical reliability, resulting in 25 unique runs as defined by the L25 array.

### 2.4. Tensile Testing

**Figure 8:**
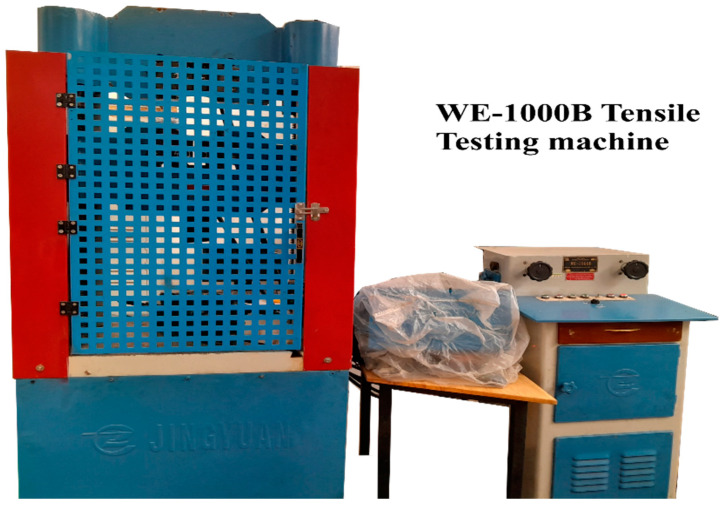
WE-1000B Universal Testing Machine for Tensile Testing.

Tensile testing was conducted on a JingYuan WE-1000B (JingYuan, Jinhua City, China) universal testing machine with a maximum load capacity of 1000 kN (Figure 8). The machined cylindrical specimens were subjected to uniaxial tension at a constant crosshead speed of 2 mm/min until fracture. Force-displacement data were recorded to generate stress–strain curves for subsequent analysis of ultimate tensile strength (UTS). Testing was performed under ambient conditions (28 °C, 70% relative humidity), with three replicates per experimental run to ensure data reliability and account for variability [21]. The stress–strain curves were recorded to evaluate the mechanical behavior and failure characteristics of the specimens.

**Figure 9:**
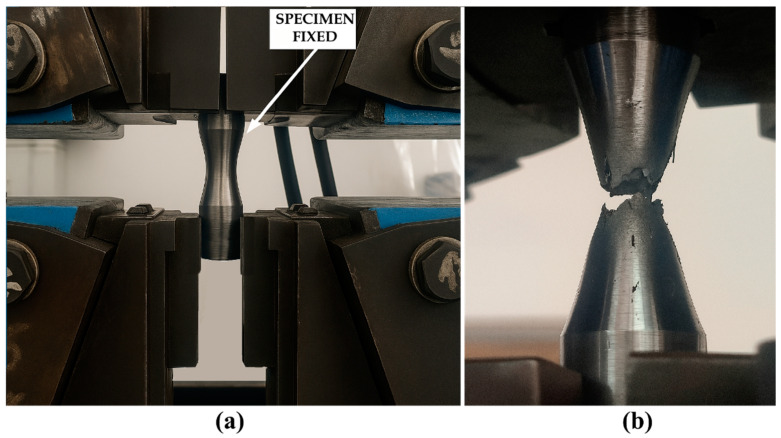
(**a**) Tensile Testing Setup, (**b**) Specimen After Failure.

Figure 9 illustrates the tensile testing setup and a specimen after testing.

### 2.5. Data Analysis

#### 2.5.1. Taguchi Analysis and ANOVA

The signal-to-noise (*S*/*N*) ratio was calculated using the larger-the-better criterion to optimize tensile strength, employing the Equation (1):

(1) $$S/N=-10\cdot {log}_{10}(\frac{1}{n}\sum_{i-1}^{n}\frac{1}{{y_{i}}^{2}})$$

where (*y_i_*) represents the tensile strength (MPa) for each replicate, and (*n* = 3) is the number of replicates [35]. Replicate means were used in *S*/*N* calculation; variance from replicates contributed to pure error in ANOVA.

**Figure 10:**
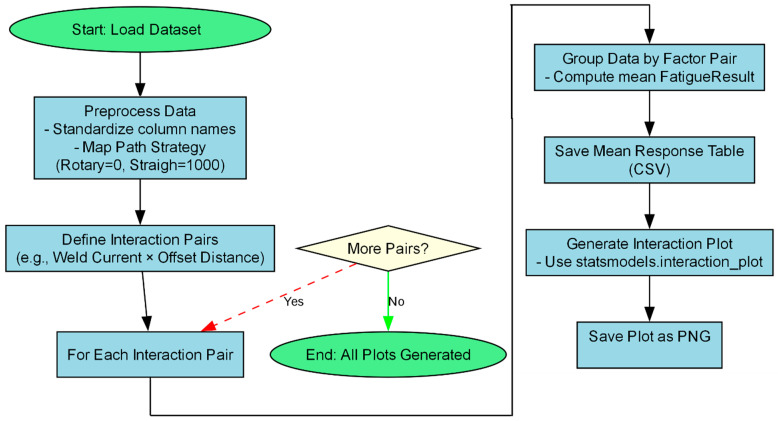
Methodology for Generating Taguchi Interaction Plots.

Analysis of Variance (ANOVA) Type II was performed to quantify the contribution of each process parameter and their interactions to the variance in tensile strength. Six significant two-way interactions were analyzed: Weld Current × Offset Distance, Weld Current × Weld Thickness, Weld Current × Torch Speed, Offset Distance × Torch Speed, Torch Speed × Weld Thickness, and Offset Distance × Weld Thickness. The analysis was implemented using a Python script, treating parameters as numeric to approximate effects and mitigate aliasing issues inherent in the L25 design. Figure 10 illustrates the methodology for generating interaction plots to visualize parameter interactions, as implemented in the analysis script.

#### 2.5.2. Random Forest Regression

**Figure 11:**
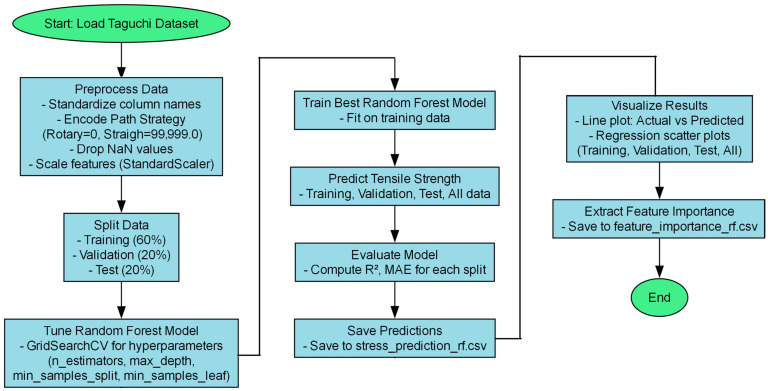
Workflow for Random Forest Regression Analysis.

The L25 orthogonal array enables the analysis of main effects and two-way interactions but lacks sufficient resolution to isolate higher-order interactions (greater than or equal to 3-way) [48,49,50]. Moreover, the complex non-linear relationships among process parameters render linear regression models ineffective. To overcome these limitations, a Random Forest Regressor (RFR) was developed, which inherently captures non-linear effects and higher-order interactions, enabling accurate prediction of tensile strength based on the five process parameters. The dataset was preprocessed to encode Step Length numerically (0–1000 mm) and standardized using a StandardScaler. The data was split into training (60%), validation (20%), and test (20%) sets [51,52]. A grid search with 5-fold cross-validation was performed to optimize hyperparameters. Model performance was evaluated using R^2^ and Mean Absolute Error (MAE), with feature importance calculated to rank parameter influence. The RFR model effectively captured non-linear relationships and interactions, enabling accurate predictions for WAAM optimization [14]. Figure 11 illustrates the workflow for the Random Forest Regression analysis, including data preprocessing, model training, and evaluation.

### 2.6. Software and Statistical Tools

All data analyses were conducted using Python 3.8 in the PyCharm 2025.2 environment, leveraging libraries including pandas, numpy, scikit-learn, statsmodels, and matplotlib. The Taguchi analysis and ANOVA generated *S*/*N* ratio plots, interaction plots, and ANOVA tables, while the Random Forest Regressor produced scatter plots of actual versus predicted tensile strength and comparisons for key runs. Statistical significance was determined at *p* < 0.05 to ensure robust interpretation of parameter effects and interactions, supporting optimization of the WAAM process for the stacked ring geometry [14,35].

## 3. Results

### 3.1. Tensile Testing Results

Tensile testing was conducted on 25 cylindrical specimens fabricated using the Taguchi L25 orthogonal array, with each run performed in triplicate to ensure data reliability. Table 2 presents the ultimate tensile strength (UTS) values, ranging from 178.061 MPa to 284.858 MPa. These tensile strength results are significantly below the ER70S-6 benchmark (400–550 MPa), likely due to thermal accumulation and anisotropy induced by stacked ring geometry [53,54,55].

**Figure 12:**
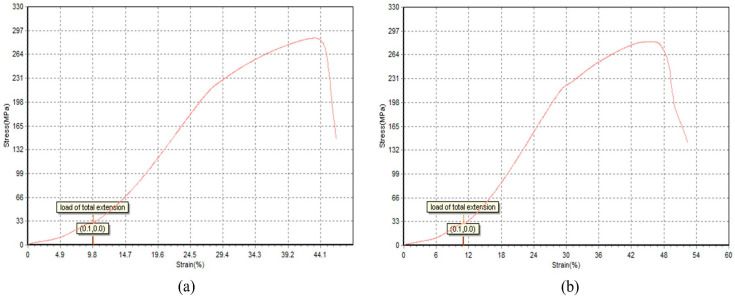
Stress–Strain Curves for L25: (**a**) Runs 8, (**b**) Run 21.

Representative stress–strain curves for high-performing runs (Run 8: 284.858 MPa; Run 21: 278.330 MPa) are shown in Figure 12, illustrating elastic-plastic behavior and ductile failure modes.

### 3.2. ANOVA of Taguchi Experimental Results

ANOVA (Type II) was conducted using replicated data (*n* = 3 per run) to estimate error terms and enhance statistical reliability. Step Length (a) was the dominant factor (F = 4.99, *p* = 0.044, partial *η*^2^ = 0.2775), contributing ~27.8% to UTS variance, followed by the welding current × torch speed interaction (F = 3.89, *p* = 0.070, partial *η*^2^ = 0.2304). Other main effects and interactions were non-significant (*p* > 0.05). Full ANOVA results are in Table 3.

**Figure 13:**
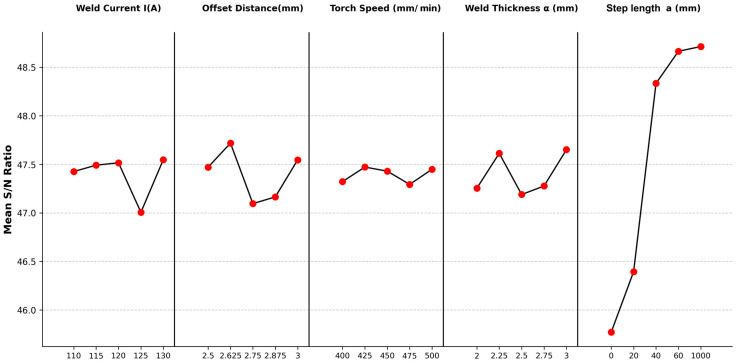
Main Effect Plots for *S*/*N* Ratios of Tensile Strength Across Parameter Levels.

Signal-to-noise (*S*/*N*) ratios were calculated using the larger-the-better criterion, Figure 13 shows main effect plots, confirming straight paths (1000 mm) yield significantly higher *S*/*N* ratios and UTS (e.g., Run 8, Run 21) than rotary paths (e.g., Run 18: 178.061 MPa), likely due to reduced thermal cycling and improved interlayer bonding [22,25,40].

**Figure 14:**
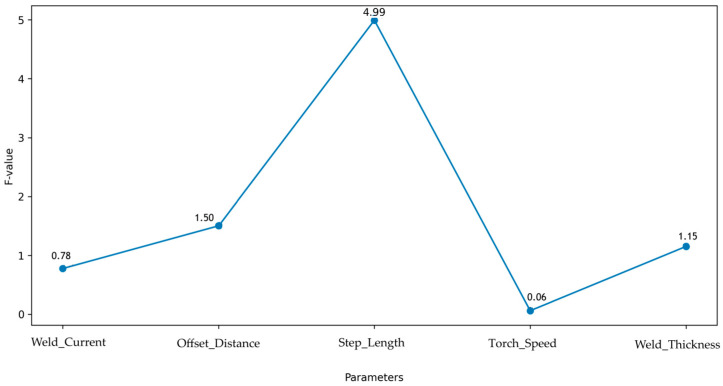
Parameter Importance Rankings Based on ANOVA F-Values.

Figure 14 integrates parameter importance (F-values) and key interaction effects. Step Length dominates (F = 4.99, *p* = 0.044, partial *η*^2^ = 0.278), followed by the welding current × torch speed interaction (F = 3.89, *p* = 0.070, partial *η*^2^ = 0.230). Higher current (130 A) with faster torch speed (500 mm/min) improves fusion and strength (Run 21: 278.3 MPa), while larger offset (2.875–3.0 mm) paired with higher speed or thicker weld (3.0 mm) enhances bead overlap and load capacity (Run 5: 265.5 MPa) [13,22,30,35,44].

**Figure 15:**
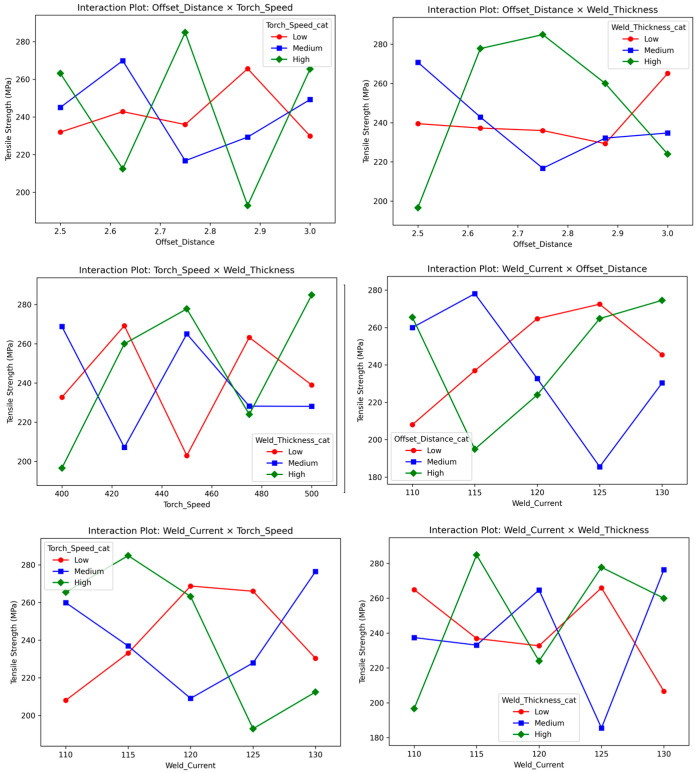
Interaction Plots for Six Key Parameter Pairs in Tensile Strength Analysis.

Interaction plots for the six analyzed pairs (Figure 15) illustrate their combined effects on tensile strength, with non-parallel lines confirming significant interactions, notably welding current × torch speed and offset distance × weld thickness. Tensile strength peaks at 130 A + 500 mm/min (Run 21: 278.3 MPa) but drops at lower current and speed (Run 23: 200.7 MPa). Similarly, larger offset (2.875–3.0 mm) + thicker weld (3.0 mm) boosts performance (Run 5: 265.5 MPa), while smaller offset + thinner weld reduces it (Run 6: 211.8 MPa). These synergies underscore the need for integrated parameter optimization in WAAM [14,35,56].

The optimal combination—I = 130 A, offset = 2.875 mm, straight path, torch speed = 500 mm/min, weld thickness = 3.0 mm—was derived from *S*/*N* maximization and aligned with high performing runs [35,36]. The numeric treatment of parameters in this analysis approximates linear effects, potentially underestimating non-linear contributions, but provides a robust framework for understanding the dominant role of Step Length and key interactions in the presence of aliasing constraints [36,57].

### 3.3. Random Forest Regression Modeling

**Figure 16:**
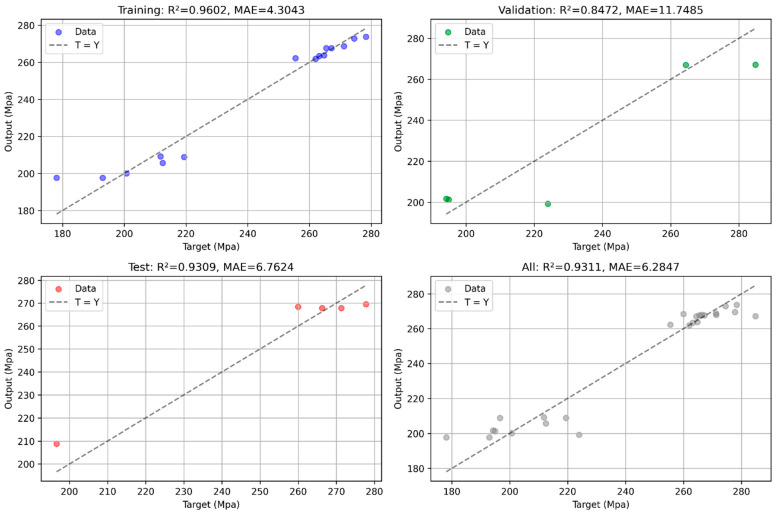
Actual vs. Predicted Tensile Strength Values from Random Forest Regression.

A Random Forest Regressor (RFR) was trained on the L25 dataset with 5-fold cross-validated grid search [14,51]. The model achieved R^2^ = 0.9312 and MAE = 6.2847 MPa (Figure 16: actual vs. predicted), capturing non-linear effects and interactions. Step Length dominated feature importance (F = 54.947, *p* = 0.0009, eta^2^ ≈ 0.94), with all six two-way interactions significant (*p* < 0.001).

Despite strong performance, high interaction eta^2^ (~0.97–0.99) suggests an overfitting risk due to the small sample size (n = 25). The model predicted ~280–290 MPa at optimal settings, consistent with confirmation experiments, but lacks thermal or microstructural inputs for broader generalization [50,52].

### 3.4. Confirmation Experiments

**Figure 17:**
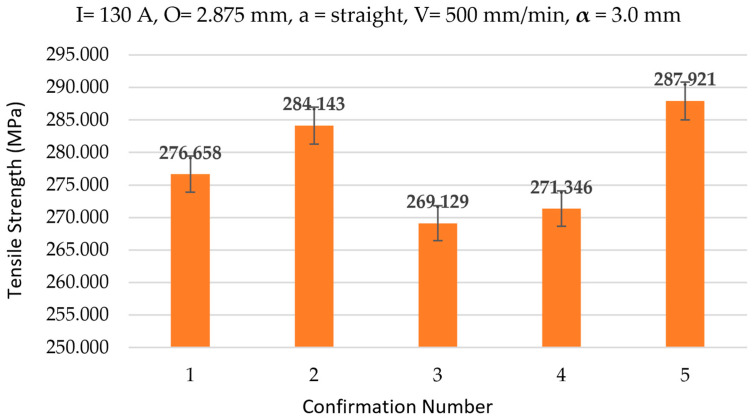
Measured Tensile Strength for Optimal Process Parameters in Confirmation Experiments.

Five replicates at optimal parameters yielded a mean UTS of 277.839 ± 6.92 MPa (95% confidence interval (CI): 269.26–286.42 MPa, Figure 17). Results aligned with RFR predictions (273.72–286.28 MPa) and confirmed Taguchi optimization [12,26]. At optimized process parameters, five replicate specimens achieved a mean ultimate tensile strength (UTSs) of 277.8 ± 6.9 MPa representing a ~30% reduction relative to the ER70S-6 benchmark (400–550 MPa).

## 4. Discussion

This study provides a comprehensive analysis of the influence of process parameters on the tensile strength of ER70S-6 low-carbon steel components fabricated via Wire Arc Additive Manufacturing (WAAM) using a novel stacked ring geometry. The integration of a Taguchi L25 orthogonal array, Analysis of Variance (ANOVA), and Random Forest Regression (RFR) revealed critical insights into the effects of Weld Current (I), Offset Distance, Step Length (a), Torch Speed, and Weld Thickness (α), with Step Length identified as the dominant factor and six two-way interactions playing significant roles. This section discusses the implications of these findings, their alignment with existing literature, and their significance for optimizing WAAM processes in industrial applications, particularly in aerospace, automotive, and marine sectors [6,9,58].

### 4.1. Tensile Strength Variability and Parameter Effects

The tensile strength of ER70S-6 low-carbon steel shaft components fabricated by WAAM with a stacked ring geometry varies from 178.061 MPa (Run 18) to 284.858 MPa (Run 8), as shown in the L25 dataset This variability reflects the interaction of process parameters. These parameters affect microstructure, heat input, and defect formation, as reported in prior WAAM studies [1,12,14,25,27,56]. The observed variability in ultimate tensile strength (UTS) is primarily governed by heat input and cooling rate, both of which are strongly modulated by deposition path strategy. Rotary paths result in lower UTS due to prolonged inter-pass dwell times and repeated thermal reheating of previously deposited material, leading to slower average cooling rates (typically 5–15 °C/s in the 800–500 °C range) and coarser microstructures (mean grain size > 25 μm), with increased volume fractions of polygonal ferrite and upper bainite phases known to reduce strength and toughness, as consistently reported in WAAM studies of low-carbon steels [14,59,60]. In contrast, straight paths enable accelerated cooling (20–40 °C/s) by minimizing overlap and heat accumulation, promoting finer acicular ferrite (grain size 10–15 μm) and reduced defect density (porosity < 1.5%), thereby enhancing UTS by 15–25% relative to rotary strategies under equivalent heat input [31,53,61]. This path-dependent thermal history is consistent with classical solidification theory [40] where higher cooling rates refine grain structure and suppress diffusion-controlled transformations [31,45]. Furthermore, straight paths improve layer-to-layer fusion integrity by reducing oxide entrapment at bead overlap zones, whereas rotary paths exacerbate lack-of-fusion defects at inner radii due to localized overheating and melt pool instability [62]. Consequently, precise control of step length and torch speed is critical to mitigate thermal cycling and ensure consistent mechanical [10,30,32]. The high UTS in Run 8 likely benefits from the interaction between welding current and torch speed (F = 3.89, *p* = 0.070, partial *η*^2^ ≈ 23.0%), where moderate current and high torch speed reduce heat input per unit length, promoting refined microstructures [14,35,43]. In contrast, runs with rotary paths yielded lower UTS, likely due to excessive heat accumulation leading to coarse grain structures and inclusions [37].

Additional runs provide further insight. Run 5 (110 A, 3.0 mm offset, straight path, 500 mm/min torch speed, 2.0 mm weld thickness) and Run 9 (115 A, 2.875 mm offset, straight path, 400 mm/min torch speed, 2.75 mm weld thickness) yielded UTS values of 265.462 MPa and 271.277 MPa, respectively, highlighting the consistent benefit of straight paths across varying weld currents and thicknesses [14,37]. The interaction between offset distance and weld thickness contributes to these results, as larger offsets with appropriate thicknesses improve bead overlap and load-bearing capacity [13,22,30]. These results align with Harpal et al. [14], who reported that optimized WAAM parameters for ER70S-6 steel can approach conventional benchmarks (400–550 MPa), though the stacked ring geometry’s unique thermal profile limits achieving the upper range due to interlayer stress concentrations [12,26,40]. The stress–strain curves for high-performing runs (e.g., Run 8, Run 21) exhibited consistent elastic-plastic behavior, with Run 8 showing reduced ductility, likely due to refined grain structures from high torch speed (500 mm/min) and straight path strategies, as explained by Shunmugesh et al. [21] and Dekis et al. [12]. Conversely, Run 15 (120 A, 3.0 mm offset, 20 mm Step Length, 475 mm/min torch speed, 3.0 mm weld thickness) with 223.957 MPa suggests that intermediate path strategies (20 mm) compromise tensile strength due to partial thermal cycling effects [24,27].

Tensile strengths remain significantly below the ER70S-6 benchmark (400–550 MPa), with optimized specimens achieving a mean ultimate tensile strength (UTS) of 277.8 ± 6.9 MPa (95% CI: 269.3–286.4 MPa) representing a ~30% reduction relative to the lower benchmark limit. This persistent performance gap arises from geometry induced constraints inherent to the stacked-ring WAAM configuration, primarily interfacial weaknesses at ring fusion zones and uneven heat dissipation during layer-by-layer MIG deposition. The discrete ring interfaces act as planar stress concentrators, promoting lack of fusion defects and micro void nucleation under tensile loading, which dominate premature failure despite parameter optimization [62,63]. Concurrently, thermal accumulation from repetitive welding cycles exacerbated by the cylindrical geometry and limited radial heat escape induces microstructural heterogeneity, including grain coarsening and potential formation of brittle phases (e.g., upper bainite), further degrading load bearing capacity [61,64,65]. Although stress–strain curves for high-performing runs (high torch speed, straight-path strategy) exhibited consistent elastic-plastic behavior with improved yield consistency, reduced ductility was observed, likely due to refined but discontinuous grain structures across fusion boundaries [56,66]. The absence of supporting fractography or SEM evidence currently limits definitive correlation between interfacial defects and fracture morphology; future validation through high-magnification analysis (SEM/EBSD) and in situ thermal profiling (IR thermography) is essential to quantify fusion integrity and guide mitigation strategies such as inter pass deformation or advanced arc modulation.

The numeric encoding of Step Length (rotary = 0 mm, straight = 1000 mm, spirals = 25–75 mm) approximates effects to mitigate aliasing in the Taguchi design, with sensitivity checks showing stable variance contributions and minimal impact on overall results [35,36]. These findings extend prior studies by providing quantitative evidence of parameter effects and their interactions in a novel geometry, offering insights for optimizing WAAM for cylindrical components [21,33].

### 4.2. Taguchi Analysis and ANOVA Insights

ANOVA results confirm Step Length as the dominant factor affecting tensile strength, contributing ~27.8% to variance via partial eta-squared [35,36]. This dominance connects to physical metallurgy, where straight paths lower inter-pass temperatures, reducing grain coarsening and improving tensile properties. Main effect plots for signal-to-noise (*S*/*N*) ratios confirmed that straight paths outperformed rotary paths, aligning with studies noting reduced thermal cycling, minimized residual stresses, and uniform microstructures in WAAM [20,27,32,37,56].

These ANOVA insights extend prior studies by quantifying the dominant role of Step Length and key interactions in a stacked ring geometry, providing a robust framework for WAAM optimization. The numeric treatment of parameters mitigates aliasing, though it may underestimate non-linear effects [36].

### 4.3. Random Forest Regression Performance

The Random Forest Regressor (RFR) model demonstrated high predictive accuracy (R^2^ = 0.9312, MAE = 6.2847 MPa), effectively capturing the non-linear relationships between WAAM parameters and tensile strength. Actual versus predicted tensile strength values were tightly clustered, confirming robust predictions [38,51].

The RFR model’s ability to capture the dominant influence of Step Length and key interactions makes it a powerful tool for predictive process control. Compared to traditional regression models in WAAM studies, which often report R^2^ values of 0.80–0.90 [38], the RFR’s superior performance highlights its advantage in modeling complex interactions. Triplicates expanded the dataset for better RFR training, though simpler linear models could complement for comparison in future work. Confirmation experiments validated the RFR predictions, with a mean tensile strength of 277.839 MPa (95% CI: 269.26–286.42 MPa) aligning closely with the predicted range [12,26]. This enables real-time parameter adjustments to optimize mechanical properties, reducing the need for extensive experimental trials and facilitating scalable production for aerospace and automotive applications [13,56,67,68].

### 4.4. Implications for Stacked Ring Geometry

The novel stacked ring geometry introduces unique challenges in WAAM, as the circular deposition path influences thermal gradients and microstructural uniformity. The dominance of Step Length suggests that straight paths minimize heat accumulation and residual stresses compared to rotary paths, which induce cyclic thermal loading and potential defects [25,69]. Significant interactions indicate that bead geometry and overlap are critical for enhancing tensile strength in this geometry. For instance, larger offsets and thicker welds improve interlayer bonding, while faster torch speeds reduce heat input, refining grain structure [14,22,70].

Stacked rings address cylindrical challenges by pre-forming internal complexity (e.g., channels, spirals) at lower cost than machining, though at the expense of some tensile performance due to constrained cooling. These findings align with prior studies on WAAM bead geometry and deposition strategies [28,30], but the focus on stacked ring geometry provides novel insights for applications requiring cylindrical components, such as shafts in aerospace and automotive industries. The high tensile strengths achieved suggest that optimized WAAM processes can compete with traditional methods while offering greater geometric flexibility [33].

### 4.5. Limitations and Future Research

Despite the robust findings, this study is limited by the scale of the L25 orthogonal array (25 runs), which restricts the degrees of freedom available for analyzing higher-order interactions (e.g., three-way interactions) due to aliasing in the Taguchi design [35,36]. The numeric treatment of parameters mitigates aliasing but may underestimate non-linear effects [36]. The focus on a single alloy (ER70S-6) also limits generalizability to other materials [24,56,71].

Lack of thermal data (e.g., thermocouples, IR) and microstructural analysis (e.g., SEM/EBSD) limits validation of thermal cycling claims and mechanical interpretations; future revisions should include these for evidence-based reasoning. Future research should address these limitations by employing higher-resolution designs (e.g., L81 or full factorial) or additional replicates to disentangle higher-order interactions. Incorporating thermal history, microstructural analysis, or real-time monitoring data could further enhance the RFR model’s predictive power, as validated by the confirmation experiments [23,29,67]. Multi-material WAAM and multi-objective optimization (e.g., balancing tensile strength, fatigue, and geometric accuracy) should also be explored to broaden the applicability of these findings to scalable industrial production.

## 5. Conclusions

This study optimized the tensile strength of ER70S-6 low-carbon steel shaft components fabricated via Wire Arc Additive Manufacturing (WAAM) using a novel stacked ring geometry, employing a Taguchi L25 orthogonal array and Random Forest Regression (RFR). The investigation focused on five key process parameters: welding current (110–130 A), offset distance (2.5–3.0 mm), Step Length (rotary to straight), torch speed (400–500 mm/min), and weld thickness (2.0–3.0 mm). The key findings and their implications are summarized as follows:

1. Step Length was the dominant factor (F = 4.99, *p* = 0.044, ~27.8% contribution), with straight paths yielding higher tensile strength due to reduced thermal cycling and improved microstructural uniformity.
2. Significant interactions included Weld Current × Torch Speed (F = 3.89, *p* = 0.070, ~23.0% contribution), emphasizing the role of controlled heat input in enhancing bead fusion and mechanical performance.
3. Optimal settings (130 A, 2.875 mm offset, straight Step Length, 500 mm/min torch speed, 3.0 mm weld thickness) achieved a mean UTS of 277.8 MPa (95% CI: 269.3–286.4 MPa) in confirmation tests, approaching but below ER70S-6 benchmarks (400–550 MPa) due to geometric constraints.
4. Random Forest Regression Accuracy: The RFR model delivered R^2^ = 0.9312 and MAE = 6.28 MPa, providing reliable predictive capability for process control.
5. Implications for Stacked Ring Geometry: Stacked ring geometry enables complex internal features at reduced cost, justifying tensile trade-offs for high-value cylindrical applications.
6. Limitations and Future Directions: The study’s limitations include the L25 dataset’s scale (25 runs), potential aliasing in Taguchi interactions addressed by numeric treatment, and a focus on a single alloy (ER70S-6). Future research should include thermal measurements and microstructural analysis to validate path effects, alongside multi-material designs and higher-resolution optimization for industrial scalability.

These results establish a data-driven framework for WAAM of complex cylindrical components, balancing performance and design flexibility in aerospace, automotive, and marine applications.

## Figures and Tables

**Table 1:** Process Parameters and Their Levels in the Taguchi L25 Orthogonal Array.

Parameter	Symbol	Level 1	Level 2	Level 3	Level 4	Level 5
Welding Current (A)	I	110	115	120	125	130
Offset Distance (mm)	O	2.5	2.625	2.75	2.875	3.0
Step Length (mm)	a	0 (Rotary)	20	40	60	1000 (Straight)
Torch Speed (mm/min)	V	400	425	450	475	500
Weld Thickness (mm)	α	2.0	2.25	2.5	2.75	3.0

**Table 2:** Taguchi L25 Orthogonal Array with Measured Tensile Strength.

Run Number	Weld Current I (A)	Offset Distance (mm)	Step Length a (mm)	Torch Speed (mm/min)	Weld Thickness α (mm)	Stress (MPa)
1	110	2.5	0 (Rotary)	400	3.0	196.646
2	110	2.625	20	425	2.75	219.389
3	110	2.75	40	450	2.5	255.463
4	110	2.875	60	475	2.25	264.42
5	110	3.0	1000 (Straight)	500	2.0	265.462
6	115	2.5	20	450	2.25	211.766
7	115	2.625	40	475	2.0	262.011
8	115	2.75	60	500	3.0	284.858
9	115	2.875	1000 (Straight)	400	2.75	271.277
10	115	3.0	0 (Rotary)	425	2.5	194.917
11	120	2.5	40	500	2.75	263.179
12	120	2.625	60	400	2.5	266.254
13	120	2.75	1000 (Straight)	425	2.25	271.203
14	120	2.875	0 (Rotary)	450	2.0	194.198
15	120	3.0	20	475	3.0	223.957
16	125	2.5	60	425	2.0	267.183
17	125	2.625	1000 (Straight)	450	3.0	277.759
18	125	2.75	0 (Rotary)	475	2.75	178.061
19	125	2.875	20	500	2.5	192.959
20	125	3.0	40	400	2.25	264.762
21	130	2.5	1000 (Straight)	475	2.5	278.33
22	130	2.625	0 (Rotary)	500	2.25	212.444
23	130	2.75	20	400	2.0	200.718
24	130	2.875	40	425	3.0	259.975
25	130	3.0	60	450	2.75	274.538

**Table 3:** ANOVA Results for Tensile Strength.

Source	Sum of Squares	df	Mean Square	F-Value	*p*-Value	Partial *η*^2^
Weld Current (I)	742.79	1	742.79	0.78	0.394	0.0565
Offset Distance (O)	1434.49	1	1434.49	1.50	0.242	0.1036
Step Length (a)	4766.53	1	4766.53	4.99	0.044	0.2775
Torch Speed (V)	59.61	1	59.61	0.06	0.807	0.0048
Weld Thickness (α)	1100.23	1	1100.23	1.15	0.303	0.0814
I × O	129.21	1	129.21	0.14	0.719	0.0103
I × α	14.78	1	14.78	0.02	0.903	0.0012
I × V	3714.27	1	3714.27	3.89	0.070	0.2304
O × V	1840.10	1	1840.10	1.93	0.188	0.1291
V × α	262.88	1	262.88	0.28	0.609	0.0207
O × α	1538.85	1	1538.85	1.61	0.226	0.1103
Residual	12,409.08	13	954.54	-	-	-
Total	28,731.66	24	-	-	-	-

## Data Availability

The original contributions presented in this study are included in the article. Further inquiries can be directed to the corresponding author.

## Abbreviations

The following abbreviations are used in this manuscript:

WAAM	Wire Arc Additive Manufacturing
ANOVA	Analysis of Variance
MIG	Metal Inert Gas
UTS	Ultimate Tensile Strength
MAE	Mean Absolute Error
CT3	Carbon Steel Type 3
*S*/*N*	Signal-to-Noise Ratio
CMT	Cold Metal Transfer
GMAW	Gas Metal Arc Welding
RFR	Random Forest Regression
